# Pooled Pharyngeal, Rectal, and Urine Specimens for the Point-of-Care Detection of *Chlamydia trachomatis* and *Neisseria gonorrhoeae* by Lay Providers in Key Population-Led Health Services in Thailand

**DOI:** 10.3390/pathogens12101268

**Published:** 2023-10-21

**Authors:** Narukjaporn Thammajaruk, Reshmie A. Ramautarsing, Akarin Hiransuthikul, Sujittra Suriwong, Waranya Tasomboon, Prasopsuk Thapwong, Atachai Phunkron, Somporn Saiwaew, Theeranat Sangpasert, Tippawan Pankam, Matthew Avery, Stephen Mills, Praphan Phanuphak, Nittaya Phanuphak

**Affiliations:** 1 Institute of HIV Research and Innovation (IHRI), Bangkok 10330, Thailand; 2 Department of Preventive and Social Medicine, Faculty of Medicine, Chulalongkorn University, Bangkok 10330, Thailand; 3 The Service Workers In Group Foundation (SWING), Bangkok 10500, Thailand; 4 Rainbow Sky Association of Thailand (RSAT), Bangkok 10240, Thailand; 5 Thai Red Cross Anonymous Clinic, Thai Red Cross AIDS Research Centre (TRCARC), Bangkok 10330, Thailand; 6 USAID/EpiC Thailand project, FHI 360, Bangkok 10330, Thailand

**Keywords:** *Chlamydia trachomatis*, *Neisseria gonorrhoeae*, CT/NG, molecular diagnostics, point-of-care detection, pooled specimens, men who have sex with men, transgender women, Thailand, Asia

## Abstract

Routine testing for *Chlamydia trachomatis* (CT) and *Neisseria gonorrhoeae* (NG) in people with heightened risk is lacking in Thailand. This study aimed to assess the performance of the Cepheid Xpert CT/NG assay, conducted by key population (KP) lay providers, for CT and NG detection on single-site and pooled specimens from the pharynx, rectum, and urine. Between August and October 2019, 188 men who have sex with men and 11 transgender women were enrolled. Participants collected urine specimens while trained KP lay providers obtained pharyngeal and rectal swabs. Compared to single-site testing with the Abbott RealTime CT/NG assay by medical technologists, the Xpert assay missed one pharyngeal NG infection out of 199 single-site specimens, giving a 93.3% sensitivity for pharyngeal NG and one missed pharyngeal NG infection out of fifty pooled specimens, giving an 88.9% sensitivity for pharyngeal NG. There was no discrepancy between the two assays for CT detection. The Cohen’s Kappa coefficient of pooled specimen testing by the Xpert was 0.93 for NG and 1 for CT when compared to single-site testing by Abbott. Implementing pooled specimen testing by KP lay providers can be a cost-saving strategy to enhance the uptake of CT/NG services for populations facing increased risk.

## 1. Introduction

Sexually transmitted infections (STIs) have a significant global impact, leading to substantial morbidity, mortality, and detrimental consequences for various aspects of health, including sexual and reproductive health, the well-being of newborns and children, and overall quality of life [1]. The World Health Organization (WHO) has set a goal of eliminating STIs as public health threats by 2030 [1]. Globally, an estimated 128.5 million new *Chlamydia trachomatis* (CT) infections and 82.4 million new *Neisseria gonorrhoeae* (NG) infections are diagnosed yearly among adults aged 15–49 years [2]. The majority of STIs are asymptomatic or exhibit mild symptoms, which pose challenges in their detection and recognition [1]. Untreated STIs can lead to significant complications such as ectopic pregnancy, infertility, chronic pelvic pain, arthritis [3], epididymitis, and urethral stricture [4], and a tenfold increase in the risk of HIV acquisition and transmission [2].

Both CT and NG possess the ability to infect multiple anatomical sites, including the pharyngeal, rectal, and urogenital sites, and can be transmitted even in the absence of noticeable symptoms [5,6,7]. Hence, there is a crucial need for effective methods to regularly screen for STIs in multiple anatomical compartments in individuals. Such screening is essential to identify asymptomatic infections, thereby preventing the development of chronic infections, complications, and further transmission. Notably, both the US Centers for Disease Control and Prevention (CDC) and the WHO recommend utilizing nucleic acid amplification tests (NAATs) over culture or gram stain methods for the diagnosis of CT/NG infections due to their superior sensitivity and specificity [4,8]. In Thailand, the 2019 updated NG guidelines recommend using gram stain for individuals with urethral, vaginal, rectal, and pharyngeal symptoms, followed by confirmatory testing when resources are available through NAATs (for urethral and vaginal samples) or culture [9]. The non-gonococcal urethritis (NGU) guidelines in Thailand, updated in 2015, recommend the use of urethral gram stain with ≥ 5 polymorphonuclear leukocyte (PMN) count/oil field or ≥30 PMN in cervical gram stain without Gram-negative intracellular diplococci for NGU diagnosis. When resources are available, NAATs are recommended for the specific diagnostic of chlamydia infection.

CT/NG screening programs in Thailand should specifically target asymptomatic individuals among populations facing increased risk. Key populations (KPs) who contribute the majority of new HIV infections in Thailand, in particular men who have sex with men (MSM) and transgender women, also bear a significant burden of CT and NG infections. Among MSM, the prevalence rates for CT and NG were found to be 21.7% and 15.5%, respectively [10]. Prevalence rates among transgender women were similarly high at 22.9% for CT and 14.3% for NG [11]. Prevalence rates were even higher among MSM living with HIV at 32.8% for CT and 23.1% for NG [12]. There is an urgent need for targeted screening and prevention in these populations.

NAATs are considered the gold standard for diagnosing CT and NG infections. However, the high cost associated with testing poses a significant barrier to their widespread implementation, especially when testing is performed across multiple anatomical compartments [13]. Nevertheless, relying solely on single-site screening would lead to a substantial proportion of CT and NG cases in KPs being missed [14]. Prior data among MSM in Thailand showed that with single-site screening, 85.9% of CT infections in the pharynx, 67.8% in the urethra, and 30.6% in the rectum would be missed, as well as 55.7% of NG infections in the pharynx, 77.4% in the urethra, and 39.6% in the rectum [10]. Similarly, among transgender women in Thailand, single-site screening would have missed 59% of CT/NG infections in the pharynx, 94% in the urethra, and 22% in the rectum [11]. These findings emphasize the importance of comprehensive screening across multiple anatomical sites to ensure the accurate detection and appropriate treatment of CT and NG infections among KPs. As established in various studies, the pooling strategy, which combines specimens from several compartments into a single test, provides a cost-effective option while maintaining accurate diagnosis [7,13,15]. Notably, Ando et al. found robust findings in pooled sample testing, with a sensitivity of 94.2% for CT and 98.3% for NG, as well as high Cohen’s Kappa coefficients of 0.945 for CT and 0.943 for NG when compared with the single-site testing with the Aptima Combo 2 assay [13].

To increase the uptake of HIV and other sexual health services among KPs, Thailand established the key population-led health services (KPLHS) model in 2015. KPLHS was designed by members of KP communities to ensure that services were tailored to the unique needs of each KP community while maintaining close ties with the public health sector [16]. Trained and qualified KP lay providers were legalized by the Thailand Ministry of Public Health to collect specimens for HIV and STI testing, perform point-of-care HIV and STI testing, inform test results to their clients, and give out oral medications as prescribed by doctors [17]. The Cepheid Xpert CT/NG assay represents a rapid point-of-care (POC) NAAT that offers highly automated processes with results available within 90 min [18], enabling prompt diagnosis and immediate initiation of patient care. Leveraging the simplicity of use and lack of need for specialized laboratory expertise, we integrated the Cepheid Xpert CT/NG testing into the KPLHS model at community-led organizations (CLOs).

In this study, we compared the performance of POC Cepheid Xpert CT/NG testing conducted by KP lay providers with those of laboratory-based Abbott RealTime CT/NG assay conducted by medical technologists for the detection of CT and NG on single-site specimens from the pharynx, rectum, and urethra. To further optimize the efficiency of POC CT/NG testing in the KPLHS context, we also compared the performance of the Cepheid Xpert CT/NG assay on pooled specimens to those of the Abbott RealTime CT/NG assay on single-site specimens. We aimed to use the study findings to guide different testing approaches to enhance CT/NG testing among KPs.

## 2. Materials and Methods

### 2.1. Study Cohort

This study analysis was part of the parental study to explore the integration of POC testing for CT and NG into KPLHS at CLOs located in three priority provinces in Thailand: Bangkok, Chonburi, and Chiang Mai. The parental study was prospectively registered on clinicaltrials.gov under the registration number NCT03580512. All participants provided their informed consent for inclusion before they participated in this study. This study was conducted in accordance with the Declaration of Helsinki, and the protocol was approved by the Institutional Review Board (IRB) of the Faculty of Medicine, Chulalongkorn University, Bangkok, Thailand (IRB. No. 728/60, with date of approval on 26 April 2018). Eligible participants included adult Thai MSM and transgender women who had engaged in at least one of the following behaviors in the past six months: unprotected anal sexual intercourse, having more than five sexual partners, prior diagnosis or treatment of bacterial STIs, and/or use of stimulant drugs. The parental study aimed to enroll four groups of participants: 300 new users of PrEP, 600 current PrEP users, 600 individuals without HIV who were not using PrEP, and 300 people living with HIV (PLHIV).

We carried out a POC CT/NG testing validation assessment at two CLOs in Bangkok, the Service Workers In Group Foundation (SWING) and the Rainbow Sky Association of Thailand (RSAT), as part of the parental study. To prepare for the study, KP lay providers from both CLOs received comprehensive training in STI counseling, STI sample collection, and POC CT/NG testing. This training was conducted through the development of training modules and administrative systems by the Institute of HIV Research and Innovation (IHRI), ensuring the competency of providers participating in this study [17]. Urine specimens and swabs from the pharynx, rectum, and neovagina (if applicable) were collected from the first 50 individuals enrolled in each of the four participant groups described above. For this study analysis, we compared the performance of POC Cepheid Xpert CT/NG testing conducted by KP lay providers with those of laboratory-based Abbott RealTime CT/NG assay at the Thai Red Cross Anonymous Clinic Laboratory, Bangkok, conducted by medical technologists for the detection of CT and NG on single-site specimens. We also compared the performance of the Cepheid Xpert CT/NG assay on pooled specimens to those of the Abbott RealTime CT/NG assay on single-site specimens collected from the same participant.

### 2.2. Specimen Collection

**Figure 1:**
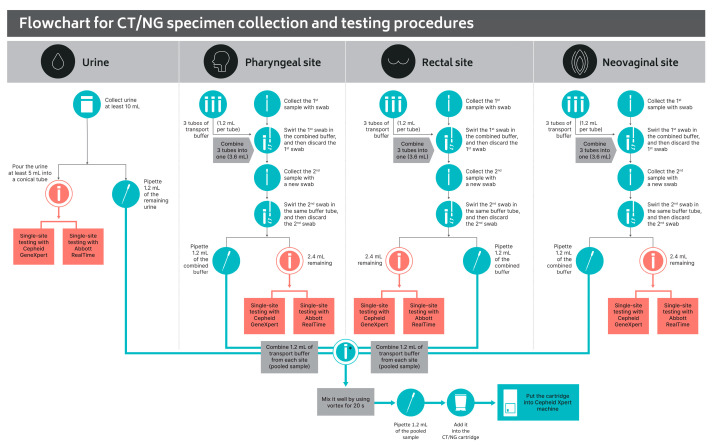
Flowchart for CT/NG specimen collection and testing procedures. CT, *Chlamydia trachomatis*; NG, *Neisseria gonorrhoeae;* * Pooled specimen preparation and subsequent testing were conducted using specimens derived from 50 PLHIV participants.

Irrespective of reported sexual routes or symptoms, collection of urine, pharyngeal swab, rectal swab, and neovaginal swab (if applicable) specimens were conducted among the participants. Swab collections were performed by trained KP lay providers, including counselors and laboratory staff, following the standard operating procedure (Figure 1).

#### 2.2.1. Urine Collection

Participants were instructed to self-collect first-catch urine specimens, ensuring a minimum volume of 10 mL. Urine specimens were then submitted to the CLO laboratory for further processing and analysis.

#### 2.2.2. Pharyngeal Collection

Prior to specimen collection, the trained KP lay providers combined three tubes of 1.2 mL specimen transport buffer from the Abbott multi-collect specimen collection kit (ref 9K12-04, Abbott Molecular Inc., Chicago, IL, USA) into a single tube, resulting in a total of 3.6 mL transport buffer in a single tube. They then used a swab from the collection kit to gently rub both tonsillar pillars and the posterior oropharynx. The swab was then swirled within the combined transport buffer and subsequently discarded. To ensure an adequate specimen for all testing procedures, the same procedures were repeated using a second swab. Finally, the specimen tube was securely capped and sent to the CLO laboratory for further processing and analysis.

#### 2.2.3. Rectal and Neovaginal Collection

The procedures for specimen collection in the rectal and neovaginal compartments were identical to those employed for pharyngeal collection, with the only difference being the anatomical site targeted. Trained providers inserted the first and second swabs into the rectum or neovagina to a depth of 2 inches and rotated them 10 times to ensure proper specimen collection.

### 2.3. Testing Procedures

#### 2.3.1. Single-Site Testing for CT and NG

The Cepheid Xpert CT/NG assay (Cepheid Inc., Sunnyvale, CA, USA) was used by trained KP lay providers for testing of single-site specimens collected from each of the four compartments: urine, pharynx, rectum, and neovagina on the same day of collection. The remaining specimens from both CLOs were stored in the refrigerator at 2–8 °C and transported in controlled-temperature containers to the Thai Red Cross Anonymous Clinic within 24 h after specimen collection for single-site testing using the Abbott RealTime CT/NG assay (Abbott m2000sp and Abbott m2000rt system, Abbott Molecular Inc., Chicago, IL, USA), conducted by trained medical technologists. All specimen collection techniques followed previous studies [19,20], and all specimen processing and storage procedures were performed in accordance with the manufacturer’s instructions to ensure specimen quality.

#### 2.3.2. Pooled Specimens Testing for CT and NG from a Single Participant

Based on previous studies [10,11], it was observed that individuals living with HIV had a higher prevalence of CT and NG infections compared to HIV-negative individuals. As a result, a pooling strategy was employed specifically for PLHIV participants. The specimen preparation for the pooling strategy was devised by the study team to ensure that the comparison results from both methods for CT and NG detection originated from the same specimen. This was important to account for any potential bias caused by the removal of organisms during the first swab, leading to a decrease in the available bacterial load for the second swab. To address this, a pooled specimen transport buffer was prepared, as indicated in Figure 1, prior to swirling the two sets of specimens into it. Pooling of specimens collected from each anatomical compartment of each PLHIV participant was conducted by trained KP lay providers in the CLO laboratory on the same day of collection. For each PLHIV participant, a volume of 1.2 mL from each of the pharyngeal, rectal, and neovaginal transport buffers was combined with 1.2 mL of fresh urine using disposable pipettes. Once combined in a conical tube, the pooled specimen was thoroughly mixed for 20 s using a vortex mixer. Subsequently, 1.2 mL of the pooled specimen was loaded into the CT/NG cartridge and inserted into the Cepheid Xpert machine by trained KP lay providers in the CLO laboratory (Figure 1).

### 2.4. Statistical Analysis

The sample size for this study was determined using the estimated one-proportion formula. The input values for this formula were based on the lowest levels of sensitivity and specificity observed for the Cepheid Xpert CT/NG assay, which were derived from the urethral compartment in males at 97.5% [21]. It should be noted that the sensitivity and specificity values for the other compartments were higher than those of the urethral compartment [21]. Considering the prevalence rates of CT and NG among MSM and transgender women in Thailand, which were reported as 27.3% for CT and 21.7% for NG [22], a sample size of 200 participants was deemed appropriate for evaluating the performance of the Cepheid Xpert CT/NG assay.

In a previous study comparing the Cepheid Xpert pooled specimen testing with the single-site specimen testing of Roche cobas 4800 assay for the detection of NG and CT, the percent agreement level was found to be 94.5% [18]. To calculate the sample size for this study, the one proportion formula was employed with a power of 90%, resulting in a sample size of 30 [23]. Since Thai MSM and transgender women living with HIV had a higher prevalence of CT (48.7% vs. 22.7%) and NG (37.7% vs. 18.3%) compared to those without HIV [22], we selectively conducted pooled testing on specimens collected from PLHIV participants. Considering these factors, a sample size of 50 PLHIV was determined to be adequate for the pooling strategy.

The performance of single-site testing and pooled specimen testing using the Cepheid Xpert assay was compared with single-site testing using the Abbott RealTime CT/NG assay. To assess the reliability and validity of these tests, various clinical performance measures were evaluated, including sensitivity, specificity, positive predictive value (PPV), negative predictive value (NPV), percent agreement, and Cohen’s Kappa coefficient. All statistical analyses were conducted using STATA 15.1 software (StataCorp LP, College Station, TX, USA). These analyses aimed to provide a comprehensive evaluation of the accuracy and agreement between the different testing methods.

## 3. Results

Of 200 participants enrolled from August to October 2019, one current PrEP user participant was excluded due to a conflict of interest involving the study staff’s participation. Among the 199 participants included in the analysis, 188 were MSM, and 11 were transgender women. No transgender women with a neovagina were recruited during this period. Hence, specimen collection was limited to three compartments: urethra, pharynx, and rectum.

### 3.1. Single-Site Testing for CT and NG

All 199 participants underwent single-site CT and NG detection using both the Cepheid Xpert CT/NG assay and the Abbott RealTime CT/NG assay. The results of these tests are summarized in Table 1. In terms of NG detection, one specimen from the pharynx was identified as positive by the Abbott assay but was not detected by the Xpert assay. Conversely, the Xpert assay detected two pharyngeal specimens and one rectal specimen that were not identified as positive for NG by the Abbott assay. Regarding CT detection, the Xpert assay detected two urine specimens and one rectal specimen that were not detected by the Abbott assay.

The comparisons of the clinical performance for the single-site testing between the Xpert assay and the Abbott assay are summarized in Table 2. For NG detection, the Xpert assay exhibited a sensitivity of 100% and specificity of 100% for urethral specimens, 93.3% and 98.9% for pharyngeal specimens, and 100% and 99.5% for rectal specimens. Regarding CT detection, the Xpert assay demonstrated a sensitivity of 100% and specificity of 99% for urethral specimens, 100% and 100% for pharyngeal specimens, and 100% and 99.4% for rectal specimens. The PPV of the Xpert assay was 71.4% (95% CI 29–96.3) for urethral CT detection and 87.5% (95% CI 61.7–98.4) for pharyngeal NG detection. The Cohen’s Kappa coefficients were 0.83 (95% CI 0.59–1) for urethral CT and 0.90 (95% CI 0.78–1) for pharyngeal NG, indicating substantial agreement. The percent agreement between the Xpert assay and the Abbott exceeded 98% for both CT and NG detection in all three compartments, supporting the reliability and validity of the Xpert assay for CT/NG detection in multiple anatomical sites.

### 3.2. Pooled Specimens Testing for CT and NG from a Single Participant

The comparison results between single-site testing and pooled specimen testing are presented in Table 3. One pharyngeal NG infection was missed when utilizing the pooled sampling strategy with the Xpert assay. To ensure clarity in interpreting these comparisons, participants who tested positive for NG or CT in one or more anatomical specimens through single-site testing using the Abbott assay (**) are reported as positive in the table.

Table 4 presents the performance of the pooled specimen testing strategy using the Cepheid Xpert assay when compared to the single-site testing using the Abbott RealTime CT/NG assay. The pooled specimen testing demonstrated a sensitivity of 88.9%, a specificity of 100% for NG detection, and a sensitivity and specificity of 100% for CT detection. With one pharyngeal NG infection missed by the pooled specimen testing, the NPV, percent agreement, and Cohen’s Kappa coefficient of the Xpert assay for NG detection decreased to 97.6% (95% CI 87.4–99.9), 98% (95% CI 94–100), and 0.93 (95% CI 0.79–1) when compared to the Abbott assay.

## 4. Discussion

We demonstrated good feasibility and performance of the Cepheid Xpert CT/NG assay, conducted by KP lay providers in Thailand, for CT and NG detection on single-site and pooled specimens from the pharynx, rectum, and urine. Compared to single-site testing by the Abbott RealTime CT/NG assay, the Xpert assay on single-site specimens missed one pharyngeal NG infection, resulting in 93.3% sensitivity for pharyngeal NG. The Xpert assay on pooled specimens also missed one pharyngeal NG infection, giving 88.9% sensitivity for pharyngeal NG. All CT infections identified by Abbott were detected by both single-site and pooled specimen testing using the Xpert. The Cohen’s Kappa coefficient of pooled specimen testing by the Xpert was 0.93 for NG and 1 for CT, compared with single-site testing by Abbott. These findings suggest that the pooled specimen strategy for POC CT/NG testing conducted by KP lay providers among KPs with increased STI risk is feasible for implementation in CLOs, potentially resulting in cost savings, reduced testing time, and alleviating staff workload, particularly for asymptomatic STI screening [7,15,20,24,25].

Additionally, we demonstrated the capability of KP lay providers to deliver STI services effectively within the KPLHS model. This encompassed various aspects such as counseling, specimen collection, result communication, point-of-care CT/NG testing, and facilitating treatment referrals, all following comprehensive training provided through the development of training modules and administrative systems by IHRI [17]. Prior to our study, Badman et al. reported a similar successful model where trained KP providers offered STI services in urban community clinics. Clients self-collected specimens for these services, while KP lay providers performed the POC CT/NG testing [7]. Importantly, our findings demonstrated the potential of KP lay providers to expand STI testing options among KPs, including the collection of specimens besides physician- and nurse-collected samples.

In Thailand, the current diagnostic methods for CT and NG mainly rely on the syndromic approach, gram strain, and culture-based techniques due to factors such as staff availability, equipment accessibility, and budget constraints [9,26]. However, these methods have limitations in diagnosing CT and NG infections, particularly where clients are either asymptomatic or present with non-specific symptoms in extragenital sites, such as sore throat, that cannot be used for diagnosis [27,28]. Additionally, culture-based testing requires specialized staff and stringent specimen handling during transportation, posing logistical challenges [29,30]. To overcome these limitations, molecular methods, specifically modular cartridge-based platforms like the Cepheid Xpert system, have proven effective in detecting both symptomatic and asymptomatic CT and NG infections [21]. However, the high cost associated with this testing approach, especially when considering the need to test multiple anatomical compartments, presents a financial barrier. To address this challenge, the specimen pooling strategy was implemented to optimize resources. By combining specimens from multiple compartments into a single test, the pooling strategy offers a solution to reduce costs while maintaining diagnostic accuracy. This approach allows for the detection of CT and NG infections in a more efficient and cost-effective manner [7,13,20,25]. Our results were consistent with the support for the pooling strategy, revealing sensitivity and specificity rates of 88.9% and 100% for NG and 100% and 100% for CT, respectively. Additionally, we observed strong Cohen’s Kappa coefficients of 0.93 for NG and 1 for CT in comparison to single-site testing.

According to the package inserts of the Cepheid Xpert CT/NG assay from the manufacturer, updated in March 2019, the sensitivity and specificity for CT and NG detection in males were only determined for urine specimens. The evaluation of extragenital compartments in males was not explicitly addressed in the package inserts. However, a meta-analysis conducted by Bristow et al. in 2019 compared the Cepheid Xpert CT/NG assay with the Aptima Combo 2 assay (Hologic, San Diego, CA, USA) for the detection of CT and NG infections in pharyngeal and rectal specimens. The study concluded that the performance of the Cepheid Xpert assay was similar to the Aptima Combo 2 assay, which is considered the reference test for extragenital specimens in reference laboratories [31]. It is important to note that our study did not utilize the Aptima Combo 2 assay as a comparator due to the limited access to this assay in Thailand. However, on 23 May 2019, the U.S. Food and Drug Administration announced the marketing of the Aptima Combo 2 assay, along with the Cepheid Xpert CT/NG assay, as the first devices for extragenital diagnostic testing of CT and NG at the pharynx and rectum [32,33]. This development filled a significant public health gap by providing additional testing options for CT and NG in extragenital sites.

Considering the Abbott RealTime assay as the reference test in our study, we observed instances of false positive results by the Xpert assay in various specimen compartments. This discrepancy may arise from the fact that the Abbott assay is not specifically recommended for extragenital specimens. Unfortunately, due to limited access to other NAATs, we were unable to conduct a third confirmatory method in our study. While caution should be exercised in interpreting false positive results, as they may lead to unnecessary treatment or psychological distress, these false positive results can serve as an opportunity for further investigation, counseling, and appropriate management to minimize the spread of STIs within the affected population.

The pharyngeal NG infection missed by the Cepheid Xpert CT/NG assay on both single-site and pooled specimens warrants further investigation. To gain insights into this result, the claimed Limit of Detection (LoD) for the Cepheid Xpert assays was examined to assess the possibility of this outcome. It was observed that the LoDs for both CT and NG in the pharyngeal specimens were higher than those for male urine and rectal specimens. Consequently, the sensitivity of the Cepheid Xpert CT/NG assay to detect CT/NG in the pharyngeal compartment was lower than in the other compartments. This discrepancy in sensitivity is attributed to both the limitations of the Cepheid Xpert machine and the lower bacterial loads typically found in the pharynx [30].

The dilution effect could also be a factor contributing to the missing of pharyngeal NG detection in the pooled sample. Badman et al. reported a sensitivity of 89.7% for NG and 90% for CT when using pooling methodology, with 7 mL urine, for the Cepheid Xpert assay [7]. Badman et al. suggested that swab specimens with low CT and NG DNA loads may be overdiluted when pooling with 7 mL of urine volume and proposed an improvement by reducing the urine volume to 1 mL in the preparation of the pooled specimen [7]. Dean et al. subsequently reported 93.2% sensitivity for NG and 98.0% for CT using 1 mL urine for pooled specimen testing and a Cohen’s Kappa coefficient of 0.925 for NG and 0.921 for CT, compared with single-site testing [25]. We demonstrated a sensitivity of 88.9% for NG and 100% for CT using 1.2 mL of urine for pooled specimen testing. Our study showed a Cohen’s Kappa coefficient of 0.93 for NG and 1 for CT.

Both the low bacterial load in the oropharyngeal compartment and the collection process for this route pose challenges. The rubbing process at the tonsillar pillars and posterior oropharynx can be difficult for non-physician staff or self-sampling as it may trigger the pharyngeal reflex in clients. To address these issues, it would be beneficial to collect the specimen from a larger surface area of the oral mucosa to reduce irritation and the occurrence of the pharyngeal reflex. Hamasuna et al. proposed the use of oral wash specimens, collected by gargling with 20 mL of saline for approximately 30 s, as an alternative specimen from the pharynx. They found that oral wash specimens were more effective in detecting pharyngeal CT infection than pharyngeal swabs [34]. Another study compared mouthwash oral-throat rinses to pharyngeal swabs and found that mouthwash specimens had a sensitivity and specificity of 72% and 99.1% for NG detection and 100% and 100% for CT detection, respectively. In comparison, using water alone had a sensitivity and specificity of 82% and 99.7% for NG detection and 100% and 99.7% for CT detection [35]. These findings suggest that gargle specimens, particularly with water or saline solution, may be an alternative option for individuals who find pharyngeal swab collection inconvenient. Introducing the collection of gargle specimens may provide a more convenient and less invasive method for detecting CT and NG infections in the pharyngeal compartment. Further research and validation studies are needed to explore the feasibility and accuracy of gargle specimens as an alternative to pharyngeal swabs in different populations and settings.

This study has certain limitations that should be acknowledged. Firstly, the small number of positive cases for CT and NG detection may have an impact on the evaluation of the pooling strategy and the performance of the Cepheid Xpert CT/NG assay. The limited sample size might affect the statistical power and generalizability of the findings. However, to overcome this challenge, we selectively conducted pooled testing on specimens collected from PLHIV participants who demonstrated a higher prevalence of CT and NG infections. Secondly, due to the unavailability of the APTIMA Combo 2 assay in Thailand, it was not used as a comparator for the Cepheid Xpert assay. Instead, the Abbott RealTime CT/NG assay was employed as the reference standard for comparison. This might limit the ability to directly compare our study findings with other previous studies. Lastly, we did not include a third assay to resolve discrepancies between the results of the Xpert assay and the Abbott assay. Therefore, there could be an overestimation of false positive results by the Xpert assay in our study.

## 5. Conclusions

This study demonstrated the feasibility of integrating Cepheid Xpert CT/NG assay into the KPLHS model in Thailand. Trained KP lay providers can perform specimen pooling and conduct POC CT/NG testing to enhance access to CT/NG test and treat services for people with heightened risk in their CLOs. Moreover, the use of pooled specimen testing from multiple body compartments shows excellent clinical performance compared to single-site testing. This practical strategy will not only help reduce testing costs but also decrease staff workload and time associated with single-site testing. We strongly recommend including the use of POC CT/NG testing on pooled specimens, which can be conducted by KP lay providers, into the national strategic plan as one key strategy to end STIs by 2030.

## Figures and Tables

**Table 1:** The testing results for CT and NG detection of the Cepheid Xpert assay compared with Abbott RealTime CT/NG assay for single-site testing.

**Single-Site Testing for NG Detection (*n* = 199)**	**Cepheid Xpert**	**Abbott RealTime**	
		**Detected** **(+)**	**Not Detected** **(-)**
Urine specimens	Detected (+)	7	0
	Not detected (-)	0	192
Pharyngeal specimens	Detected (+)	14	2
	Not detected (-)	1	182
Rectal specimens	Detected (+)	15	1
	Not detected (-)	0	183
**Single-site testing for CT detection (*n* = 199)**	**Cepheid Xpert**	**Abbott RealTime**	
		**Detected** **(+)**	**Not detected** **(-)**
Urine specimens	Detected (+)	5	2
	Not detected (-)	0	192
Pharyngeal specimens	Detected (+)	7	0
	Not detected (-)	0	192
Rectal specimens	Detected (+)	27	1
	Not detected (-)	0	171

CT, *Chlamydia trachomatis*; NG, *Neisseria gonorrhoeae*.

**Table 2:** Clinical performances of the Cepheid Xpert assay compared with the Abbott RealTime CT/NG assay for single-site testing.

Specimen Type	Sensitivity (%, 95% CI)	Specificity (%, 95% CI)	PPV (%, 95% CI)	NPV (%, 95% CI)	Percent Agreement (%, 95% CI)	Cohen’s Kappa Coefficient (95% CI)
**Urethral**						
NG	100 (59–100)	100 (98.1–100)	100 (59–100)	100 (98.1–100)	100	1
CT	100 (47.8–100)	99 (96.3–99.9)	71.4 (29–96.3)	100 (98.1–100)	99 (97.6–100)	0.83 (0.59–1)
**Pharyngeal**						
NG	93.3 (68.1–100)	98.9 (96.1–99.9)	87.5 (61.7–98.4)	99.5 (97–100)	98.5 (96.8–100)	0.90 (0.78–1)
CT	100 (59–100)	100 (98.1–100)	100 (59–100)	100 (98.1–100)	100	1
**Rectal**						
NG	100 (78.2–100)	99.5 (97–100)	93.8 (69.8–100)	100 (98–100)	99.5 (98.5–100)	0.97 (0.9–1)
CT	100 (87.2–100)	99.4 (96.8–100)	96.4 (81.7–99.9)	100 (97.9–100)	99.5 (98.5–100)	0.98 (0.94–1)

CT, *Chlamydia trachomatis*; NG, *Neisseria gonorrhoeae*; 95% CI, 95% confidence interval; PPV, positive predictive value; NPV, negative predictive value.

**Table 3:** Comparison of test results between pooled specimen testing by the Cepheid Xpert assay and single-site testing by the Abbott RealTime CT/NG assay.

**NG** **(*n* = 50)**	**Cepheid Xpert for Pooled Specimen Testing**	**Abbott RealTime for Single-Site Testing**	
		Detected ** (+)	**Undetected (-)**
	Detected (+)	8	0
	Undetected (-)	1	41
**CT** **(*n* = 50)**	**Cepheid Xpert for Pooled Specimen Testing**	**Abbott RealTime for Single-Site Testing**	
		Detected ** (+)	**Undetected (-)**
	Detected (+)	16	0
	Undetected (-)	0	34

CT, *Chlamydia trachomatis*; NG, *Neisseria gonorrhoeae*. ** Participants who tested positive for NG or CT in one or more anatomical specimens through single-site testing using the Abbott assay.

**Table 4:** Comparison of clinical performance between pooled specimen testing by the Cepheid Xpert assay and single-site testing by the Abbott RealTime CT/NG assay.

Detection	Sensitivity (%, 95% CI)	Specificity (%, 95% CI)	PPV (%, 95% CI)	NPV (%, 95% CI)	Percent Agreement (%, 95% CI)	Cohen’s Kappa Coefficient (95%CI)
NG	88.9 (51.8–99.7)	100 (91.4–100)	100 (63.1–100)	97.6 (87.4–99.9)	98 (94–100)	0.93 (0.79–1)
CT	100 (79.4–100)	100 (89.7–100)	100 (79.4–100)	100 (89.7–100)	100	1

CT, *Chlamydia trachomatis*; NG, *Neisseria gonorrhoeae*; 95% CI, 95% confidence interval; PPV, positive predictive value; NPV, negative predictive value.

## Data Availability

This study did not produce or scrutinize supplementary data beyond the information presented in this publication. Data sharing is not applicable to this article.
